# Association of Serum Lipid Profile With Diabetic Retinopathy Severity in Adults With Type 2 Diabetes Mellitus: A Cross-Sectional Study From Eastern India

**DOI:** 10.7759/cureus.115052

**Published:** 2026-08-23

**Authors:** Lalhmingmawii C, Marianus Deepak Lakra, Vatsala Kunwar, Rashmi Tirkey

**Affiliations:** 1 Ophthalmology, Regional Institute of Ophthalmology, Rajendra Institute of Medical Sciences, Ranchi, IND

**Keywords:** adults, diabetic retinopathy, diabetic retinopathy severity, dyslipidemias, india, ldl cholesterol, serum triglycerides, type 2 diabetes mellitus (t2dm)

## Abstract

Background

Dyslipidemia is a frequent metabolic disorder, common in type 2 diabetes mellitus. Serum lipid parameters have increasingly become the focus of interest as potentially modifiable risk factors for the development and progression of diabetic retinopathy. Hence, the present study was designed to assess the association between serum lipid parameters and the severity of diabetic retinopathy among adults with type 2 diabetes mellitus attending a tertiary care center.

Methods

A cross-sectional study was conducted among 98 adults with type 2 diabetes mellitus. Diabetic retinopathy was classified using the Early Treatment Diabetic Retinopathy Study (ETDRS) classification. Fasting blood glucose, postprandial blood glucose, and serum lipid profile were estimated. Association of serum lipid parameters with severity of diabetic retinopathy was assessed, and logistic regression was performed to determine the factors independently associated with diabetic retinopathy.

Results

Serum lipid abnormalities were significantly related to diabetic retinopathy severity. Triglycerides had the highest association with disease severity, and total cholesterol, high-density lipoprotein (HDL) cholesterol, and low-density lipoprotein (LDL) cholesterol also differed significantly between retinopathy grades. Hypercholesterolemia and hypertriglyceridemia were significant on univariate analysis, while hypertriglyceridemia was the only independent risk factor after multivariable adjustment.

Conclusion

Serum lipid abnormalities were associated with the severity of diabetic retinopathy in adults with type 2 diabetes mellitus. Higher triglyceride and total cholesterol levels were observed across categories of diabetic retinopathy, and hypertriglyceridemia remained independently associated with the presence of diabetic retinopathy in multivariable analysis. These findings support further prospective studies to clarify the temporal and potentially modifiable relationship between dyslipidemia and diabetic retinopathy.

## Introduction

Diabetes mellitus is a major global public health concern, with a rapidly rising prevalence and significant contributions to cardiovascular, renal, neurological, and ocular morbidity. Diabetic retinopathy (DR) is one of the primary microvascular complications and is a major cause of preventable visual impairment and blindness in working-age adults globally. Although DR care and retinal imaging have improved, the disease continues to grow in parallel with the increasing prevalence of diabetes, and it is crucial to identify modifiable risk factors that might delay DR onset and progression [1,2]. DR is chiefly caused by chronic hyperglycemia, which leads to oxidative stress, inflammation, endothelial dysfunction, and blood-retinal barrier breakdown, causing progressive retinal microvascular damage [3]. While the duration of diabetes and poor glycemic control are known factors that affect DR, they don't necessarily account for the significant differences in disease severity even among people with similar glycemic control, so other systemic factors are thought to play a role in the progression of DR [4,5].

Dyslipidemia is a frequent metabolic disorder, common in type 2 diabetes mellitus (T2D), which is defined by elevated triglycerides, increased low-density lipoprotein cholesterol (LDL-C), and decreased high-density lipoprotein cholesterol (HDL-C). Therefore, serum lipid parameters have increasingly become the focus of interest as potentially modifiable risk factors for DR development and progression [6,7]. But the association between serum lipid profile and DR is not fully understood. Several clinical studies have shown strong relationships between its level of total cholesterol, triglycerides and LDL cholesterol and the severity of retinopathy or diabetic macular edema (DME); however, others have found it to have weak or inconsistencies after taking into account traditional risk factors [8,9]. The apparent disparities in study populations, diagnostic criteria, ethnicity, glycemic status and lipid-lowering therapy may in part explain these conflicting findings and the generalizability of available evidence is limited in eastern India despite the escalating burden of diabetes in this region [10].

With these unknowns, additional studies are warranted to explore the relationship between serum lipid abnormalities and DR severity in different clinical settings. Understanding this association better could help to identify patients at a higher risk for more advanced stages of retinal disease earlier, and highlight the need for thorough metabolic management in addition to optimal glycemic control. Hence, the present study was designed to assess the association between serum lipid parameters and the severity of DR among adults with T2D attending a tertiary care center.

## Materials and methods

This was a cross-sectional study conducted at the Regional Institute of Ophthalmology, Rajendra Institute of Medical Sciences (RIMS), Ranchi, Jharkhand, India, from June 2024 to November 2025, following approval from the RIMS Institutional Ethics Committee (Approval letter no. 118). Written informed consent was obtained from all participants prior to data collection. Consecutive adult patients (age ≥ 18 years) with a diagnosis of T2D who were attending the ophthalmology outpatient department were recruited. Patients with retinal vascular disease other than DR, media opacity that precluded fundus evaluation, previous vitreoretinal surgery, retinal disease unrelated to diabetes, pregnancy, and incomplete clinical or biochemical records were excluded. The minimum sample size was calculated using the formula for estimating a single-population proportion, with a 95% confidence level (CI), a desired absolute precision of 10%, and a reported prevalence of DR in people with diabetes from a recent systematic review and meta-analysis [5]. A total sample size of about 96 subjects was obtained, so 98 subjects were selected by the consecutive sampling technique.

Demographic and clinical data like age, sex, duration of diabetes mellitus, and treatment modality were obtained by a predesigned case record form. A thorough eye examination was conducted on all participants, including best corrected visual acuity measurement using a Snellen chart, slit lamp biomicroscopy, intraocular pressure measurement, and dilated fundus examination. DR was classified using the Early Treatment Diabetic Retinopathy Study (ETDRS) classification, and clinically significant macular edema (CSME) was evaluated using standard ETDRS diagnostic criteria and included in the analysis based on the worst eye [11]. After an overnight fast, venous blood glucose samples were drawn for the estimation of fasting blood glucose, postprandial blood glucose, and serum lipid profile (total cholesterol, triglycerides, HDL-C, and LDL-C) using standardized enzymatic methods in the institutional central laboratory. Hypercholesterolemia was defined as total cholesterol ≥200 mg/dL, hypertriglyceridemia as triglycerides ≥150 mg/dL, elevated LDL-C as LDL-C ≥130 mg/dL, and low HDL-C as <40 mg/dL in men and <50 mg/dL in women using the National Cholesterol Education Program Adult Treatment Panel III (NCEP ATP III) guidelines [12].

All data were entered into Microsoft Excel (Microsoft Corp., Redmond, WA, USA) and analysed using IBM SPSS Statistics for Windows, Version 26 (Released 2019; IBM Corp., Armonk, New York, United States). Continuous variables were presented as mean ± standard deviation and categorical variables were summarized by their frequency and percentages. Association of serum lipid parameters with severity of DR was assessed. Logistic regression was performed to determine the factors independently associated with DR. A p-value <0.05 was considered statistically significant.

## Results

A total of 98 patients with T2D were included in the analysis. Demographic, clinical, and biochemical baseline parameters are shown in Table 1.

**Table 1:** Baseline characteristics of the study participants mg/dL, milligram per deciliter; SD, Standard Deviation; OHA, Oral hypoglycemic drugs; HDL, High-density lipoprotein; LDL, Low-density lipoprotein.

Variable		Overall (n=98)
Gender	Male, n (%)	71 (72.4)
	Female, n (%)	27 (27.6)
Treatment	OHA, n (%)	84 (85.7)
	Insulin, n (%)	14 (14.3)
Age (years), mean ± SD		57.50 ± 9.37
Duration of diabetes (years), mean ± SD		8.07 ± 3.87
Fasting blood glucose (mg/dL), mean ± SD		119.24 ± 41.97
Postprandial blood glucose (mg/dL), mean ± SD		186.78 ± 65.28
Total cholesterol (mg/dL), mean ± SD		218.29 ± 41.31
Triglycerides (mg/dL), mean ± SD		199.57 ± 62.57
HDL cholesterol (mg/dL), mean ± SD		49.63 ± 8.79
LDL cholesterol (mg/dL), mean ± SD		111.59 ± 28.80

**Figure 1:**
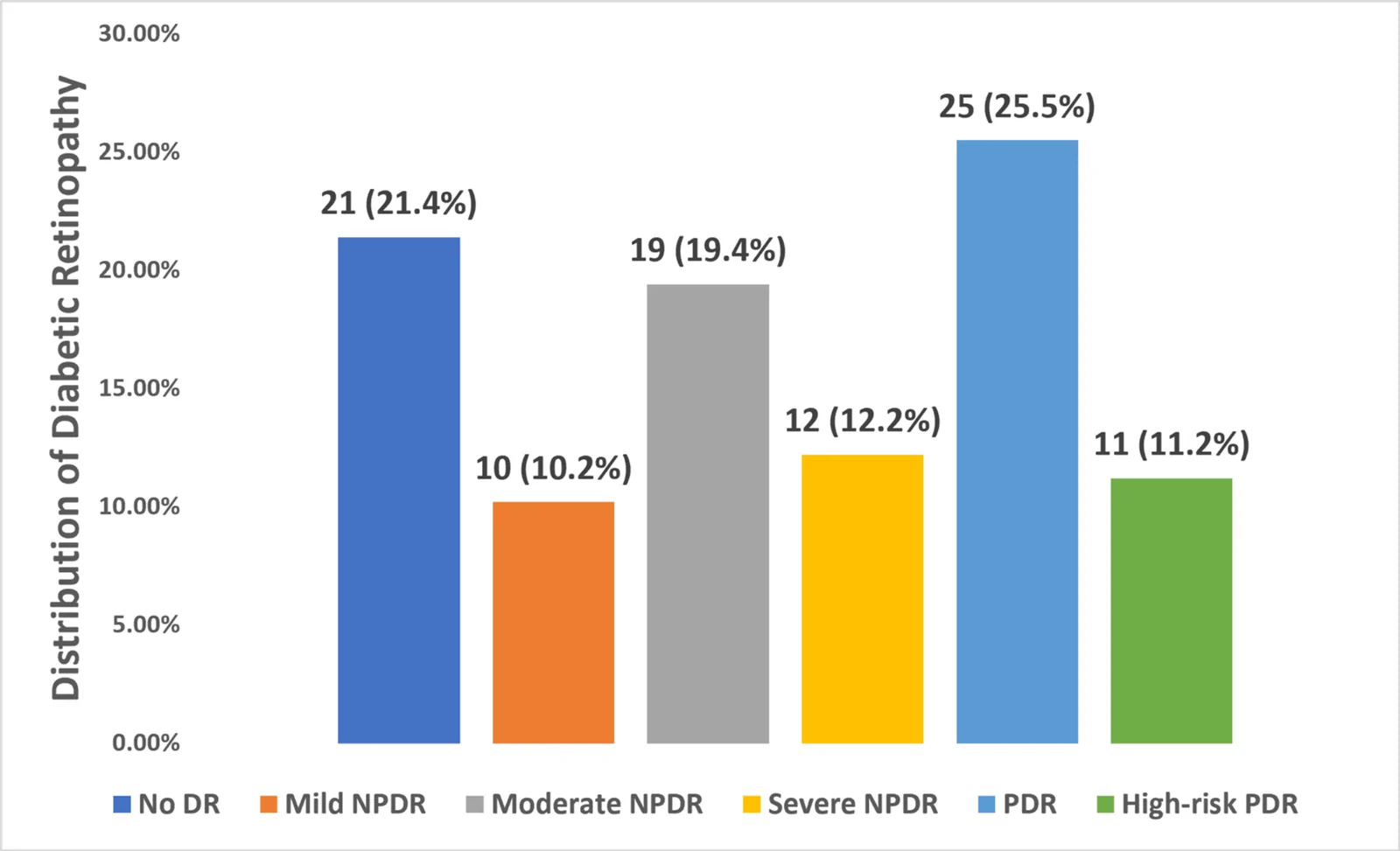
Distribution of diabetic retinopathy severity (n=98) DR, Diabetic retinopathy; PDR, Proliferative diabetic retinopathy; NPDR, Non-proliferative diabetic retinopathy.

Most of the participants were detected as having DR and the most common stage was proliferative DR. CSME was present in 21 of 77 participants with DR (27.3%) and in none of the 21 participants without DR (Fisher's exact p=0.006) (Figure 1 and Table 2).

**Table 2:** Distribution of clinically significant macular edema (CSME) Yates-corrected χ² test = 5.626, Fisher's exact test p-value: 0.006. DR, Diabetic retinopathy; PDR, Proliferative diabetic retinopathy; NPDR, Non-proliferative diabetic retinopathy.

Retinopathy status	CSME present	CSME absent	Total
No DR	0 (0.0%)	21 (100.0%)	21
Any DR (NPDR/PDR)	21 (27.3%)	56 (72.7%)	77
Total	21 (21.4%)	77 (78.6%)	98

There was significant difference in the serum lipid parameters among the different stages of DR. Triglyceride, total cholesterol, HDL-C and LDL-C concentrations differed significantly across DR severity categories (Table 3).

**Table 3:** Serum lipid parameters according to diabetic retinopathy (DR) severity *One-way ANOVA; mg/dL, milligram per deciliter; SD, Standard deviation; HDL, High-density lipoprotein; LDL, Low-density lipoprotein; PDR, Proliferative diabetic retinopathy; NPDR, Non-proliferative diabetic retinopathy; HR-PDR, High-risk proliferative diabetic retinopathy.

Variable (Mean ± SD)	No DR (n=21)	Mild NPDR (n=10)	Moderate NPDR (n=19)	Severe NPDR (n=12)	PDR (n=25)	HR-PDR (n=11)	F Value	P value*
Total cholesterol (mg/dL)	184.62 ± 33.05	230.40 ± 23.08	221.00 ± 33.11	230.92 ± 53.30	225.60 ± 45.66	236.45 ± 23.70	4.459	0.001
Triglycerides (mg/dL)	141.43 ± 28.19	168.00 ± 19.18	185.63 ± 27.04	265.00 ± 41.43	241.96 ± 67.30	195.64 ± 62.18	16.780	<0.001
HDL cholesterol (mg/dL)	51.10 ± 9.99	57.30 ± 10.57	47.89 ± 8.25	45.83 ± 4.67	49.04 ± 7.99	48.36 ± 7.79	2.479	0.037
LDL cholesterol (mg/dL)	116.81 ± 21.46	123.80 ± 14.22	128.89 ± 30.80	96.33 ± 22.31	100.60 ± 29.11	102.27 ± 34.66	4.052	0.002

Hypercholesterolemia and hypertriglyceridemia were significant factors associated with DR on univariate analyses while low HDL-C and high LDL-C were not (Table 4).

**Table 4:** Association between dyslipidemia and diabetic retinopathy (DR) *Univariate analysis: any DR vs no DR; OR, Odds ratio; CI, Confidence interval; TC, Total cholesterol; TG, Total triglyceride; HDL, High-density lipoprotein; LDL, Low-density lipoprotein.

Lipid Abnormality	No DR (n=21)	Any DR (n=77)	Unadjusted OR (95% CI)	P value
Hypercholesterolemia	7 (10.3%)	61 (89.7%)	7.63 (2.64–22.04)	<0.001
Hypertriglyceridemia	7 (9.2%)	69 (90.8%)	17.25 (5.38–55.35)	<0.001
Low HDL*	7 (33.3%)	14 (66.7%)	0.44 (0.15–1.30)	0.134
Elevated LDL*	18 (26.9%)	49 (73.1%)	0.29 (0.08–1.08)	0.054

Multivariable adjustment showed that hypertriglyceridemia was the only lipid abnormality that remained independently associated with the presence of DR (adjusted OR 13.51, 95% CI 2.68-66.67; p=0.002). The wide CI indicates limited precision in the effect estimate (Table 5).

**Table 5:** Multivariable logistic regression for factors associated with diabetic retinopathy (DR) OR, Odds ratio; CI, Confidence interval; TC, Total cholesterol; TG, Total triglyceride; HDL, High-density lipoprotein; LDL, Low-density lipoprotein; FBS, Fasting blood sugar; PPBS, Post-prandial blood sugar; mg/dL, milligram per deciliter.

Variable	Adjusted OR	95% CI	P value
Hypercholesterolemia	3.18	0.68–14.93	0.142
Hypertriglyceridemia	13.51	2.68–66.67	0.002
Low HDL	0.28	0.05–2.00	0.163
Elevated LDL	0.19	0.03–1.30	0.091
Age (per year)	0.98	0.90–1.08	0.741
Male sex	0.50	0.07–3.33	0.473
Duration of diabetes (per year)	1.07	0.84–1.36	0.612
FBS (per mg/dL)	1.01	0.97–1.05	0.645
PPBS (per mg/dL)	1.01	0.98–1.03	0.572

## Discussion

In our study, serum lipid abnormalities were found to be significantly related to DR among the patients with T2D. DR was found in 78.6% of the 98 participants, with the highest prevalence being the proliferative DR. Serum triglyceride and total cholesterol concentrations differed significantly across DR severity categories, and hypertriglyceridemia was the only lipid abnormality that remained independently associated with the presence of DR after adjustment for age, sex, duration of diabetes, glycemic status, and other lipid parameters (adjusted OR 13.51, 95% CI 2.68-66.67, P=0.002). Hypercholesterolemia, however, was not statistically significant after the multivariable adjustment, and neither HDL-C nor LDL-C was independently associated with DR. This association of increasing total cholesterol and more severe DR seen in this study is supported by several clinical studies [13,14]. In our study, the mean total cholesterol was higher in the high-risk proliferative DR group (236.5 mg/dL) than that in the non-DR group (184.6 mg/dL), by about 28%. The higher total cholesterol concentrations observed among participants with more advanced DR are consistent with previous reports suggesting an association between dyslipidemia and retinal vascular abnormalities.

The most pronounced differences across DR severity categories were observed for triglycerides. Mean triglyceride concentrations were lowest in participants without DR (141.43 mg/dL) and highest in those with severe NPDR (265.00 mg/dL; P<0.001). Triglyceride levels remained elevated in the proliferative stages, although the pattern across increasing DR severity was not strictly linear. In contrast, neither HDL-C nor LDL-C was independently associated with DR in the current study, although these lipids have been linked to DR progression in recent studies and meta-analyses [15,16]. Experimental studies have further shown that excess triglycerides promote oxidative stress, endothelial dysfunction, inflammation, and breakdown of the blood-retinal barrier, which accelerates retinal ischemia and neovascularization [17]. There was a significant difference in HDL-C levels across ETDRS grades; however, HDL-C was not independently associated with DR after adjustment for the variables included in the multivariable model. Similarly, LDL-C differed significantly across ETDRS severity categories in the overall analysis but was not independently associated with DR in the multivariable model. Notably, the pattern of LDL-C across severity categories was non-monotonic, with lower mean LDL-C levels in severe NPDR and proliferative stages than in some earlier stages. This finding should therefore not be interpreted as evidence of a progressive relationship between increasing LDL-C levels and DR severity. The observed pattern may reflect sampling variability or residual confounding, particularly because lipid-lowering therapy was not recorded. These findings are consistent with previous studies reporting less consistent associations between conventional lipid fractions and DR after accounting for other metabolic factors [18,19]. Furthermore, paradoxical or inverse associations between lipid fractions and DR have been reported, suggesting that circulating LDL-C and HDL-C concentrations may not fully reflect alterations in retinal lipid metabolism [20].

Large randomized trials (Fenofibrate Intervention and Event Lowering in Diabetes (FIELD) and Action to Control Cardiovascular Risk in Diabetes Eye substudy (ACCORD Eye)) showed that fenofibrate was effective in slowing the progression of DR and reducing the need for laser treatment, despite its relatively modest effects on conventional serum lipid levels, suggesting that its beneficial effects may involve mechanisms beyond lipid lowering [21,22]. These trial findings provide clinically relevant context for the association between hypertriglyceridemia and DR observed in our study. However, given the cross-sectional design and the absence of information on lipid-lowering therapy in our participants, the present findings should not be interpreted as evidence that triglyceride lowering itself prevents the development or progression of DR.

The major strengths of this study are the use of the same grading method for DR (ETDRS), the measurement of the entire lipid profile, and the use of multivariable analysis to explore whether the observed lipid associations persisted after adjustment for the measured demographic and clinical variables. Moreover, the study gives the current data from a tertiary care center located in the eastern part of the country where there is paucity of published data regarding dyslipidaemia and DR. This study has several limitations. First, its cross-sectional design does not allow causal or temporal relationships between lipid abnormalities and DR to be established. The relatively small sample size and single-center setting may also limit the generalizability of the findings and may have introduced referral bias towards more advanced disease. An important limitation was the absence of information on lipid-lowering therapy, including statins and fibrates, which could substantially alter measured lipid concentrations and confound the observed associations with DR. Fenofibrate, in particular, has been reported to influence DR outcomes through mechanisms that may not be fully explained by changes in conventional serum lipid concentrations. In addition, HbA1c was not available; therefore, fasting blood sugar and post-prandial blood sugar were used as measures of glycemic status but may not adequately reflect long-term glycemic exposure. Data on BMI, blood pressure or hypertension, renal function, and relevant lifestyle factors were also unavailable. Residual confounding by lipid-lowering therapy and these unmeasured metabolic and clinical factors therefore cannot be excluded, and the observed associations should be interpreted cautiously. Furthermore, the multivariable logistic regression included several covariates despite only 21 participants being in the no-DR group, which may have reduced the stability and precision of the estimates and increased the possibility of model overfitting. The wide CI for the association between hypertriglyceridemia and DR (adjusted OR 13.51, 95% CI 2.68-66.67) further indicates uncertainty regarding the magnitude of this association. Larger prospective, multicenter studies incorporating comprehensive measures of long-term glycemic control, lipid-lowering therapy, and other relevant metabolic and clinical factors are needed to clarify the relationship between dyslipidemia and the development and progression of DR.

## Conclusions

Our study demonstrated that serum lipid abnormalities were significantly associated with the severity of DR in patients with T2D. Higher serum levels of total cholesterol and triglycerides were associated with greater severity of DR, and hypertriglyceridemia was the only lipid abnormality that remained independently associated with the presence of DR after adjustment for the demographic and clinical variables included in the multivariable model. However, no independent association was observed between DR and HDL-C or LDL-C. These findings suggest that hypertriglyceridemia may have a stronger association with DR than other conventional lipid parameters. Given the cross-sectional design of the study, these findings should be interpreted as associations and do not establish a causal relationship between dyslipidemia and the development or progression of DR. Further large-scale prospective studies incorporating important metabolic and treatment-related factors are needed to clarify the relationship between serum lipid abnormalities and DR and to determine whether lipid-lowering interventions influence long-term ocular outcomes.
